# KRAS mutation-induced upregulation of PD-L1 mediates immune escape in human lung adenocarcinoma

**DOI:** 10.1007/s00262-017-2005-z

**Published:** 2017-04-27

**Authors:** Nan Chen, Wenfeng Fang, Zhong Lin, Peijian Peng, Juan Wang, Jianhua Zhan, Shaodong Hong, Jiaxing Huang, Lin Liu, Jin Sheng, Ting Zhou, Ying Chen, Hongyu Zhang, Li Zhang

**Affiliations:** 1 State Key Laboratory of Oncology in South China, Department of Medical Oncology, Sun Yat-Sen University Cancer Center, 651 Dongfeng Road East, Guangzhou, Guangdong People’s Republic of China; 2 Collaborative Innovation Center for Cancer Medicine, Sun Yat-sen University Cancer Center, Guangzhou, Guangdong People’s Republic of China; 3 Department of Medical Oncology, the Fifth Affiliated Hospital of Sun Yat-sen University, Zhuhai, Guangdong People’s Republic of China

**Keywords:** KRAS, PD-L1, PD-1, Lung adenocarcinoma

## Abstract

It was reported that PD-L1 expression was correlated with genetic alterations. Whether PD-L1 was regulated by mutant Kirsten rat sarcoma viral oncogene homolog (KRAS) in non-small-cell lung cancer (NSCLC) and the underlying molecular mechanism were largely unknown. In this study, we investigated the correlation between PD-L1 expression and KRAS mutation and the functional significance of PD-1/PD-L1 blockade in KRAS-mutant lung adenocarcinoma. We found that PD-L1 expression was associated with KRAS mutation both in the human lung adenocarcinoma cell lines and tissues. PD-L1 was up-regulated by KRAS mutation through p-ERK but not p-AKT signaling. We also found that KRAS-mediated up-regulation of PD-L1 induced the apoptosis of CD3-positive T cells which was reversed by anti-PD-1 antibody (Pembrolizumab) or ERK inhibitor. PD-1 blocker or ERK inhibitor could recover the anti-tumor immunity of T cells and decrease the survival rates of KRAS-mutant NSCLC cells in co-culture system in vitro. However, Pembrolizumab combined with ERK inhibitor did not show synergistic effect on killing tumor cells in co-culture system. Our study demonstrated that KRAS mutation could induce PD-L1 expression through p-ERK signaling in lung adenocarcinoma. Blockade of PD-1/PD-L1 pathway may be a promising therapeutic strategy for human KRAS-mutant lung adenocarcinoma.

**Electronic supplementary material:**

The online version of this article (doi:10.1007/s00262-017-2005-z) contains supplementary material, which is available to authorized users.

## Introduction

Lung cancer remains the leading cause of cancer-related death worldwide [1]. Non-small-cell lung cancer (NSCLC) accounts for approximately 80% of all lung cancers [2]. Lung adenocarcinoma, as the most common pathologic type of NSCLC, is often accompanied with oncogenic driver mutation [3, 4]. Driver mutations like epidermal growth factor receptor mutation (EGFR) and echinoderm microtubule associated protein like 4 and anaplastic lymphoma kinase (EML4-ALK) fusion are highly sensitive to their corresponding tyrosine kinase inhibitors (TKIs) [5]. Kirsten rat sarcoma viral oncogene homolog (KRAS) is the most common driver mutation in lung adenocarcinoma patients of non-Asian ethnicity [6]. The prevalence of KRAS mutation in lung adenocarcinoma in Asian and Western patients is approximately 11 and 26%, respectively [7]. In addition, KRAS mutation is usually mutually exclusive with other major driver mutations such as EGFR and ALK [8]. Recent studies show that patients with KRAS-mutant lung cancer respond poorly to EGFR-TKIs [9, 10]. Furthermore, KRAS mutation is a negative predictor of the efficacy of chemotherapy [11]. Until now, the more effective treatment strategies are urgently needed for KRAS-mutant NSCLC.

Immune checkpoint molecules, programmed death-1 receptor (PD-1, CD279) and programmed death ligand 1 (PD-L1, B7-H1 or CD274) play an important role in tumor immune escape [12]. Recent development of immune checkpoint inhibitors such as anti-PD-1 antibody and anti-CTLA-4 antibody has shown promising results in specific subset of NSCLC patients [13, 14]. Some studies reported that high PD-L1 expression was correlated with EGFR mutation and ALK fusion protein in NSCLC [15–17]. Thus, exploring the association between PD-L1 expression and driver mutations and determining the effect of immune checkpoint inhibitors on oncogene addicted NSCLC are crucial in clinical practice. However, whether PD-L1 is regulated by KRAS and the underlying molecular mechanisms are largely unknown. Moreover, the effect of blocking PD-L1/PD-1 axis on T cells and NSCLC cells and its potential clinical value in KRAS-mutant NSCLC have not been fully elucidated.

In this study, we investigated the correlation between PD-L1 and KRAS mutation and the regulatory mechanism. We also tried to explore whether blocking the PD-1/PD-L1 axis could be a novel therapeutic option for lung adenocarcinoma with KRAS mutation.

## Materials/patients and methods

### Cell lines and cell culture

Human NSCLC cell lines H460, H1299, H2228, H292 and H1993 were obtained from the American Type Culture Collection. Immortalized human lung bronchial epithelial cell (Beas-2B), EKVX, Beas-2B-vector, Beas-2B-KRAS-G12D and Beas-2B-KRAS-WT cells were generously provided by Prof. Liang Chen (National Institute of Biological Sciences, China). H358 was kindly provided by Prof. Mengfeng Li (Department of Microbiology, Zhongshan School of Medicine, Sun Yat-sen University, China). H358, H460 and EKVX are the KRAS-mutant NSCLC cell lines. H1299 is the N-RAS-mutant lung adenocarcinoma cell line. H2228 is the lung adenocarcinoma cell line with EML4-ALK fusion. H292 and H1993 are NSCLC cell lines with EGFR/ALK/KRAS wild-type (WT). Beas-2B-KRAS-G12D, Beas-2B-KRAS-WT and Beas-2B-vector are the Beas-2B cells stably transfected with KRAS G12D mutant, KRAS wild-type and control plasmid, respectively. H2228 and H358 were cultured in RPMI-1640 complete growth medium supplemented with 10% fetal bovine serum and antibiotics (10,000 U/ml penicillin and 10 μg/ml streptomycin). Other cell lines were grown in DMEM complete medium.

### Western blot analysis and quantitative real-time PCR

Western blot analysis was done as previously reported [15]. The primary antibodies for PD-L1 (E1L3N™), RAS (D2C1), mutant KRAS (G12D Mutant Specific) (D8H7), phospho-p44/42MAPK (ERK1⁄2) (Thr202⁄Tyr204), p44/42MAPK (ERK1/2), phosphor-AKT (Ser473), AKT and GAPDH were purchased from Cell Signaling Technology (CST). Quantitative real-time PCR experiments were performed as previous described [18].

### Surface staining of PD-L1 with flow cytometry

Suspension cells (Beas-2B-vector, Beas-2B-KRAS-G12D and Beas-2B-KRAS-WT) were stained with PD-L1 (E1L3N, Rabbit mAb, PE Conjugated) or the corresponding isotype control (DA1E, Rabbit mAb IgG, PE Conjugated) (CST, Danvers, MA). The surface expression of PD-L1 were detected by flow cytometry and analyzed with FlowJo 7.6.1 software [15].

### Immunofluorescence

H358, H1993, Beas-2B-KRAS-G12D and Beas-2B-vector cells were fixed and blocked before addition of the primary antibodies including PD-L1 (E1L3N™, Rabbit mAb) or mutant KRAS (G12D mutant specific, D8H7, Rabbit mAb) at 4 °C overnight. Then cells were incubated with secondary antibody (Alexa Fluor 488 or 555 donkey anti-rabbit IgG [H+L], Life Technologies, LA) for 1 h. The detailed protocol was described in previous report [15].

### KRAS siRNA, inhibitors and cell viability analysis

KRAS siRNAs were purchased from Ribobio Corporation (Guangzhou, China). The target sequence of KRAS siRNA #1 and siRNA #2 are CGAATATGATCCAACAATA and CAAGAGGAGTACAGTGCAA, respectively. Beas-2B-KRAS-G12D and H358 cells were transiently transfected with KRAS siRNAs using Lipofectamine^®^ RNAiMAX_Reagent (Invitrogen) for 48 h. ERK1/2 inhibitor (SCH772984) and AKT1/2/3 inhibitor (MK-22062HCL) were purchased from Selleckchem (Houston, USA). Beas-2B-KRAS-G12D cells or H358 cells were exposed to climbing doses of ERK and AKT inhibitors for 72 h. The viability of the cells was tested with CCK8 kit (Cell Counting Kit-8, Dojindo.Co, Japan). Recombinant humanized anti-PD-1 antibody, Pembrolizumab (MK-3475, Keytruda) was from Merck Sharp & Dohme Corp (Whitehouse Station, NJ08889, USA).

### Co-culture system and apoptosis assay with flow cytometry

Dendritic cells and cytokine-induced killer cells (DC-CIK) were kindly provided by Prof. Jianchuan Xia (Department of Biotherapy, Sun Yat-sen University Cancer Center, China) [19, 20]. The protocol of acquiring DC-CIK was described in our previous report [21]. Beas-2B-KRAS-G12D, Beas-2B-vector, H358 and EKVX cells were seeded into 12-well plates at a density of 1.0 × 10^5^ cells/well, respectively. The acquired DC-CIK were added into co-culture system with Beas-2B-KRAS-G12D, Beas-2B-vector,H358 or EKVX cells at the ratio of 1:1, respectively. Next, the DC-CIK/Beas-2B-KRAS-G12D, DC-CIK/H358 and DC-CIK/EKVX co-culture system were treated with mock, Pembrolizumab (500 μg/ml), ERK1/2 inhibitor (100 nM/L) or AKT inhibitor (1.0 μM/L), respectively. After 48 h, suspended DC/CIK cells were removed from the adherent Beas-2B-KRAS-G12D, H358 or EKVX cells in the cell culture plate with pipet. Then, after washing with PBS, suspended DC/CIK cells were stained with anti-human CD3 FITC antibody (OKT-3, 11-0037, Affymetrix eBioscience) for 15 min. Next, after washing, DC-CIK cells were stained with Annexin V-APC and 7-AAD for 15 min with Apoptosis Detection kit (KGA1023-1026, KeyGEN, Nanjing, China) [21]. The apoptotic cells of CD3+ T cells detected by flow cytometry (Beckman Gallios, Beckman-Coulter, Inc. USA) were defined as Annexin V-APC-positive cells (both 7-AAD-negative and -positive) from the gate of CD3-positive cells. The example of gating strategy is presented in supplementary Fig. 1.

**Fig. 1:**
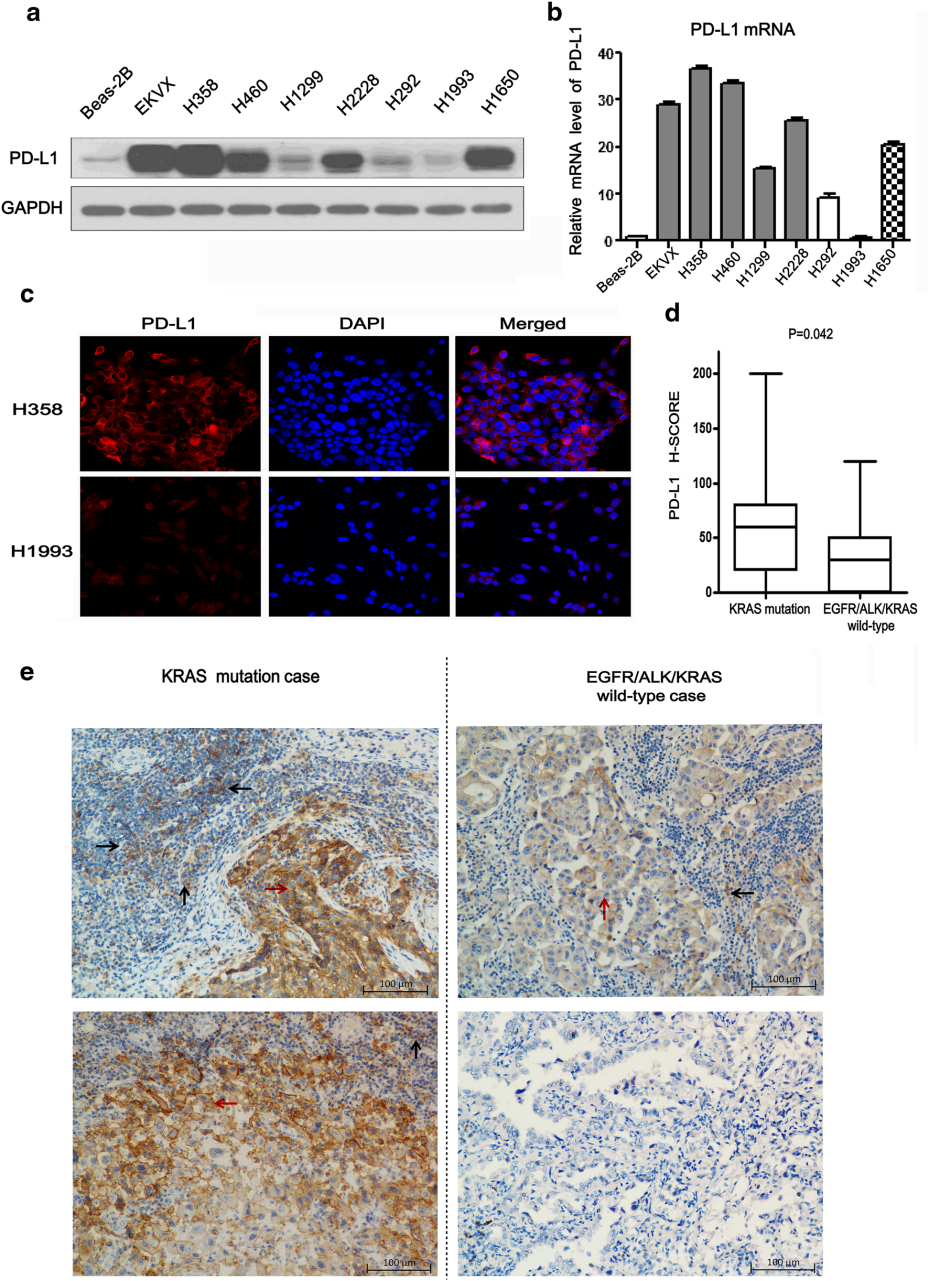
PD-L1 expression correlated with KRAS mutation. **a** The protein expression levels of PD-L1 were detected by western blot in different NSCLC cell lines and Beas-2B. GAPDH was used as loading reference. **b** The relative expression levels of PD-L1 mRNA were detected by real time PCR in above-mentioned cells. **c** The localization of PD-L1 (*red signal*) in H358 and H1993 cell lines were shown by immunofluorescence counterstained with DAPI (*blue signal*). Original magnification: ×600. **d** Significant association of PD-L1 H-SCORE with KRAS status (19 cases of lung adenocarcinoma with KRAS mutation and 38 cases of lung adenocarcinoma with EGFR/ALK/KRAS wild-type). Data are presented as *box plots*, and *P* values were determined with the Wilcoxon rank-sum test. **e** Representative images of PD-L1 immunohistochemical staining in two KRAS-mutant cases with strong staining intensity (*left panels*) and two EGFR/ALK/KRAS wild-type cases with weak staining intensity (*right panels*). *Black arrows* indicate tumor-infiltrating immune cells. *Red arrows* indicate tumor cells. Original magnification: ×400

### Real time cells survival analysis

The survival rates of KRAS-mutant tumor cells like H358 or EKVX cells were dynamically monitored in real time by the xCELLigence system (E-plate, Roche) which could exclude the interference of suspended DC-CIK. Firstly, 96-well E-plate with 50 μl of complete growth medium in each well was tested in the incubator to establish a background reading. Next, tumor cells (1.0 × 10^4^ cells/well) were seeded into 96-well E-plates for approximately 20 h followed by addition of DC-CIK (50 μl/well) into the E-plates at a DC-CIK: tumor cells ratio of 1:1. Finally, an additional 50 μl/well of the complete medium containing different drugs such as vehicle, Pembrolizumab (500 μg/ml), ERK1/2 inhibitor (100 nM/L) and Pembrolizumab (500 μg/ml) plus ERK1/2 inhibitor (100 nM/L) were added into the DC-CIK/H358 or DC-CIK/EKVX co-culture system, respectively. H358 cells alone were meanwhile treated with vehicle, Pembrolizumab (500 μg/ml) and ERK1/2 inhibitor (100 nM/L) as the control groups. Cell index values were monitored every 15 min from each well of E-plate and presented as the dynamic cell growth curves [21, 22].

### Patients and clinical data

Our study prospectively enrolled 216 newly diagnosed NSCLC patients who all underwent genomic analysis of EGFR, ALK and KRAS from April 2013 to December 2014 in Sun Yat-sen University Cancer Center (SYSUCC). This study was approved by the Institutional Review Board of SYSUCC and written informed consent was obtained before specimens were collected. The specimens were from surgical resection tissue or biopsies of the untreated patients. KRAS and EGFR mutation status were tested using real-time PCR. ALK rearrangements were detected by fluorescence in situ hybridization. Excluding the patients with EGFR mutation and ALK fusion, the remaining 69 patients were pathologically diagnosed as lung adenocarcinoma with EGFR/ALK wild-type. Among them, there were 19 patients harboring KRAS mutation. Patients’ baseline characteristics were collected including gender, age, smoking status, tumor differentiation and staging. Pathologic or clinical staging was determined according to the cancer staging manual (7th edition) of American Joint Committee on Cancer. Using “MatchIt” package of R programming language, baseline characteristics of patients were balanced matching between KRAS mutation group and EGFR/ALK/KRAS wild-type group by propensity matching score analysis [23]. Subsequently, statistic analysis has been carried out for 19 patients with KRAS mutation matched with 38 out of 50 patients with EGFR/ALK/KRAS wild-type. Finally, PD-L1 expression in the tissue of 57 patients after matching was detected by immunohistochemistry.

### Immunohistochemistry

Immunohistochemical staining was performed using PD-L1 rabbit antibody (E1L3N™, CST; dilution 1:200) overnight at 4 °C. Immunoreactivity was detected using the DAKO ChemMateEnVision method according to the manufacturer’s instructions. Two pathologists blinded to patients’ information independently assessed expression of PD-L1. Semi-quantitative H score (H-SCORE) was determined by multiplying the percentage of positively stained cells by an intensity score (0, absent; 1, weak; 2, moderate; and 3, strong) and ranged 0–300.

### Statistical analysis

The SPSS software (version 19.0) was used for statistical analysis. After matching with “MatchIt” package of R programming language, the differences of gender, smoking status, tumor differentiation, staging between KRAS mutation group and EGFR/ALK/KRAS wild-type group were examined by the Pearson Chi-square test and the difference of age between the two groups was examined by two independent samples’ *t* test. Wilcoxon rank-sum test was used to compare the H-SCORE of PD-L1 staining between KRAS mutation and EGFR/ALK/KRAS wild-type group. Representative results from three independent experiments were shown in this study. Numerical data were presented as the mean ± standard deviation of the mean (SD). The *P* values between two experimental groups were tested by two-tailed Student’s *t* test and *P* values less than 0.05 were considered significant.

## Results

### PD-L1 expression was correlated with KRAS mutation in lung adenocarcinoma

In order to investigate the association between PD-L1 expression and KRAS mutation status, we conducted experiments in human lung adenocarcinoma cell lines and tissue. We found that the protein levels of PD-L1 in endogenous KRAS-mutant NSCLC cell lines (EKVX, H358, H460), EML4-ALK fusion cell line (H2228), and EGFR 19-exon deletion mutation cell line (H1650) were significantly higher than that in EGFR/ALK/KRAS wild-type cell lines (H292, H1993), N-RAS-mutant cell line (H1299), or lung bronchial epithelial cell line (Beas-2B cell) (Fig. 1a). Similar results of PD-L1 mRNA level were also observed in the above-mentioned cell lines (Fig. 1b). Sub-cellular localization of PD-L1 protein was detected in H358 and H1993 cells using immunofluorescence. PD-L1 showed stronger membrane localization in H358 cells (red fluorescence), compared with that in H1993 cells (Fig. 1c). Next, we detected PD-L1 expression on tumor cells and tumor-infiltrating immune cells in patients’ lung adenocarcinoma tissue by immunohistochemistry. Baseline characteristics of patients including gender, age, smoking history, tumor differentiation and stage were balanced matching between KRAS mutation group and EGFR/ALK/KRAS wild-type group by propensity matching score analysis [24] (Table 1). KRAS-mutant cases tended to have higher intensity of PD-L1 staining than EGFR/ALK/KRAS wild-type cases. PD-L1 immunoreactivity was detected mainly on the cytomembrane or slightly in the cytoplasm (or both) of tumor cells or tumor-infiltrating immune cells (Fig. 1e). The median of H-SCORE was significantly higher in KRAS mutation cases than in EGFR/ALK/KRAS wild-type cases (60 vs. 30, *P* = 0.042) (Fig. 1d). The results implied that PD-L1 expression was associated with KRAS mutation in lung adenocarcinoma.

**Table 1:** Baseline characteristics of lung adenocarcinoma patients

Characteristics	Total, *n* = 57	KARS mutation, *n* = 19 (% with kras mutation	EGFR/ALK/KRAS wild type, *n* = 38 (% with wild type)	*P* value after matching
Gender				0.838
Female	17	6 (31.6%)	11 (28.9%)	
Male	40	13 (68.4%)	27 (71.1%)	
Age, years				0.838
<60	25	9 (47.4%)	16 (42.1%)	
≥60	32	10 (52.6%)	22 (57.9%)	
Smoking history				0.703
Smokers	34	12 (63.2%)	22 (57.9%)	
Non-smokers	23	7 (36.8%)	16 (42.1%)	
Tumor differentiation				0.764
Poor	22	7 (36.8%)	15 (39.5%)	
Moderate	31	10 (52.6%)	21 (55.3%)	
Well	4	2 (10.5%)	2 (5.3%)	
Stage				1.000
I	27	9 (47.4%)	18 (47.4%)	
II	12	4 (21.1%)	8 (21.1%)	
IIIA	9	3 (15.8%)	6 (15.8%)	
IIIB–IV	9	3 (15.8%)	6 (15.8%)	

### Over-expression or knockdown of KRAS altered PD-L1 expression

**Fig. 2:**
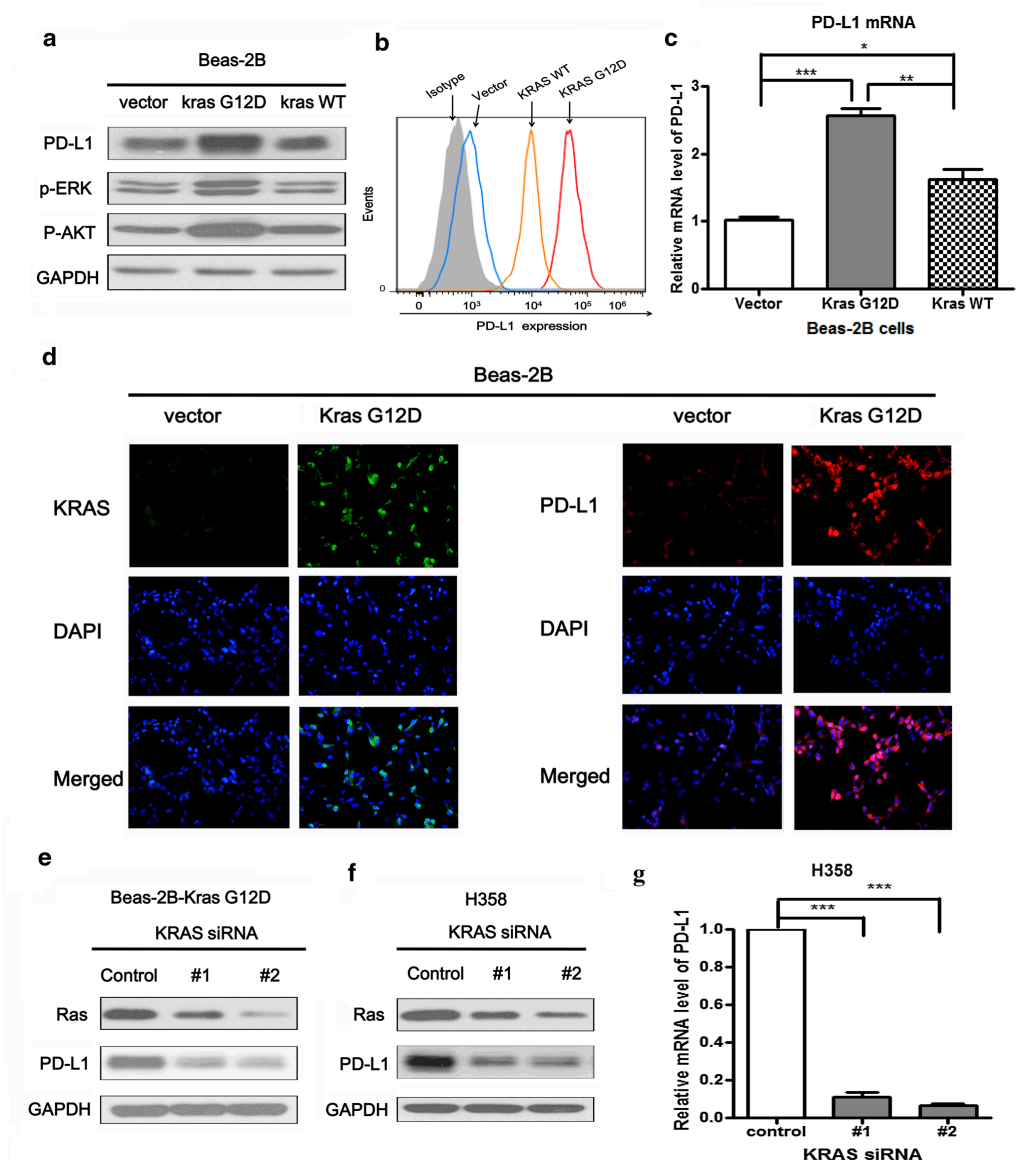
Both over-expression and knockdown of KRAS regulated PD-L1 expression. **a** The protein expression level of PD-L1, p-ERK and p-AKT in Beas-2B cells stably transfected with KRAS-G12D mutation, KRAS wild-type and control plasmid, respectively. **b** The surface expression level of PD-L1 were detected by flow cytometry in Beas-2B-KRAS-G12D, Beas-2B-KRAS-WT, Beas-2B-vector and isotype control cells. **c** The relative mRNA level of PD-L1 in Beas-2B-KRAS-G12D, Beas-2B-KRAS-WT and Beas-2B-vector cells. **d** The localization of PD-L1 (*red signal*) and KRAS (*green signal*) in Beas-2B-vector and Beas-2B-KRAS-G12D cells were shown by immunofluorescence counterstained with DAPI (*blue signal*). Original magnification: ×200. **e**, **f** The protein expression level of Ras and PD-L1 were detected by western blot in Beas-2B-KRAS G12D and H358 cells which were both transiently transfected with two loci KRAS-siRNAs for 48 h. **g** The relative mRNA level of PD-L1 in H358 cells which were transiently transfected with two loci KRAS-siRNAs for 48 h. Representative data of three independent experiments are shown. *P < 0.05; **P < 0.001; ***P < 0.0001

Firstly, we found the expression of PD-L1 was significantly higher in Beas-2B-KRAS-G12D cells compared with Beas-2B-vector cells. However, the expression of PD-L1 in Beas-2B-KRAS-WT cell was not obviously increased compared with control cells. Likely, the phosphorylated expression of ERK1/2 and AKT were activated by mutant KRAS-G12D but not wild-type KRAS (Fig. 2a). Similarly, flow cytometric analysis showed the surface expression of PD-L1 in Beas-2B cells with KRAS G12D mutation was higher than that in Beas-2B cells with KRAS wild-type and vector control (Fig. 2b). The induction of PD-L1 by over-expression of exogenous KRAS was further confirmed at the mRNA level by PCR. The higher level of PD-L1 mRNA was observed in Beas-2B-KRAS-G12D cells than that in Beas-2B-vector and Beas-2B-KRAS-WT cells (Fig. 2c). Immunofluorescence staining also showed the association between KRAS and PD-L1 protein expression. Increased expression of KRAS and PD-L1 was observed on the cytomembrane of Beas-2B cells with KRAS G12D over-expression (Fig. 2d). In contrast, knocking down KRAS expression using two loci KRAS siRNAs decreased the expression level of Ras in Beas-2B-KRAS G12D cells. PD-L1 was also significantly down-regulated following KRAS knockdown (Fig. 2e). Similarly, in H358 cells, the protein expression and mRNA level of PD-L1 were both significantly down-regulated following the knockdown of KRAS by siRNAs (Fig. 2f, g). Taken together, our results demonstrated that over-expression of exogenous KRAS induced PD-L1 expression and knocking down KRAS reduced PD-L1 expression. PD-L1 expression level was positively correlated with KRAS mutation.

### KRAS up-regulated PD-L1 through p-ERK but not p-AKT signaling

**Fig. 3:**
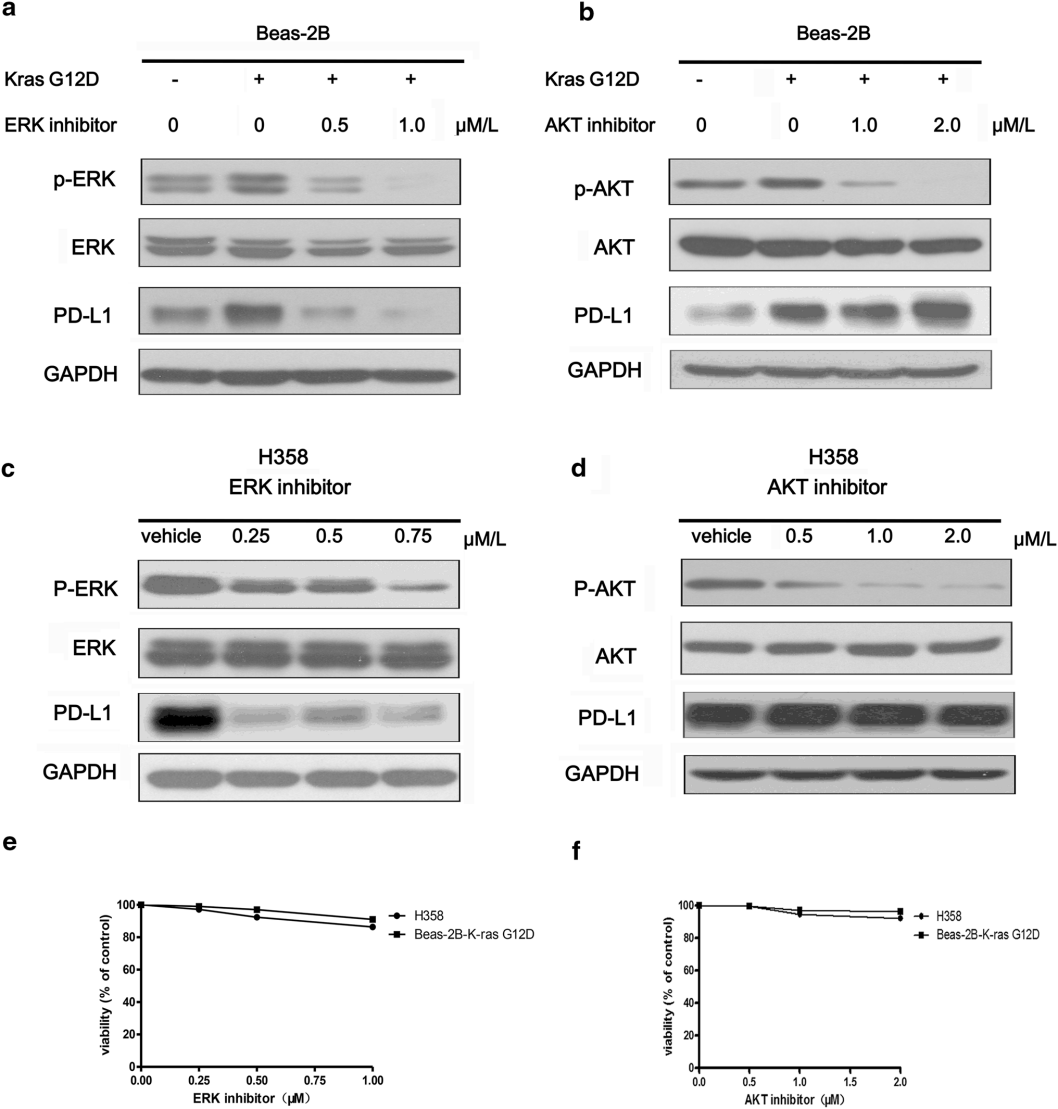
KRAS regulated PD-L1 through p-ERK but not p-AKT signaling. **a** The protein expression level of p-ERK, ERK, PD-L1, GAPDH in Beas-2B-vector cells and Beas-2B-KRAS-G12D cells which were treated with 0, 0.5, 1.0 μM ERK1/2 inhibitor (SCH772984) for 48 h. **b** The protein expression level of p-AKT, AKT, PD-L1, GAPDH in Beas-2B-vector cells and Beas-2B-KRAS G12D cells which were treated with 0, 1.0, 2.0 μM/L AKT inhibitor (MK-2206 2HCL) for 48 h. **c** The protein expression level of p-ERK, ERK, PD-L1 and GAPDH in H358 cell which were treated with 0, 0.25, 0.5, 0.75 μM/L ERK1/2 inhibitor (SCH772984) for 48 h. **d** The protein expression level of p-AKT, AKT, PD-L1 and GAPDH in H358 cell which were treated with 0, 0.5, 1.0, 2.0 μM AKT inhibitor (MK-2206 2HCL) for 48 h. Vehicle represented PBS. **e**, **f** Cell viability of H358 cells and Beas-2B-KRAS G12D cells were detected with CCK8 kit when they were treated with climbing doses of ERK 1/2 inhibitor (0, 0.25, 0.75, 1.0 μM/L) and AKT inhibitors (0, 0.5. 1.0, 2.0 μM/L) for 72 h. The experiments were repeated three times. Representative data are shown

In order to investigate how KRAS regulate PD-L1 expression, firstly, we examined the two key downstream proteins of Ras pathway (p-ERK and p-AKT) by western blot. Figure 2a showed both p-ERK1/2 and p-AKT were activated by KRAS G12D mutation. Then, we used the inhibitors of ERK1/2 and AKT to block the downstream pathways of KRAS in Beas-2B cells with exogenous expression of KRAS G12D mutation and H358 cell with endogenous expression of KRAS G12C mutation. We found that ERK1/2 inhibitor (SCH772984) inhibited the p-ERK, which further resulted in the decrease of PD-L1 expression (Fig. 3a, c). However, AKT inhibitor (MK-2206 2HCL) could effectively suppress p-AKT, but did not alter the expression level of PD-L1 (Fig. 3b, d). To investigate whether PD-L1 is regulated through the mechanism of specific signaling pathway or the effect of cell viability, we performed the viability analysis of H358 and Beas-2B-KRAS G12D cells to find that the indicated concentration of ERK inhibitor and AKT inhibitor only weakly inhibited the viability of the two cells (Fig. 3e, f). Thus, PD-L1 regulated by ERK1/2 signaling is dependent on signaling pathway mechanism. Collectively, KRAS mutation induced PD-L1 expression through p-ERK1/2 signaling pathway but not p-AKT pathway.

### KRAS could induce the apoptosis of T cells through PD-1/PD-L1 axis and blocking PD-1/PD-L1 could reverse the apoptosis of T cells in co-culture system

**Fig. 4:**
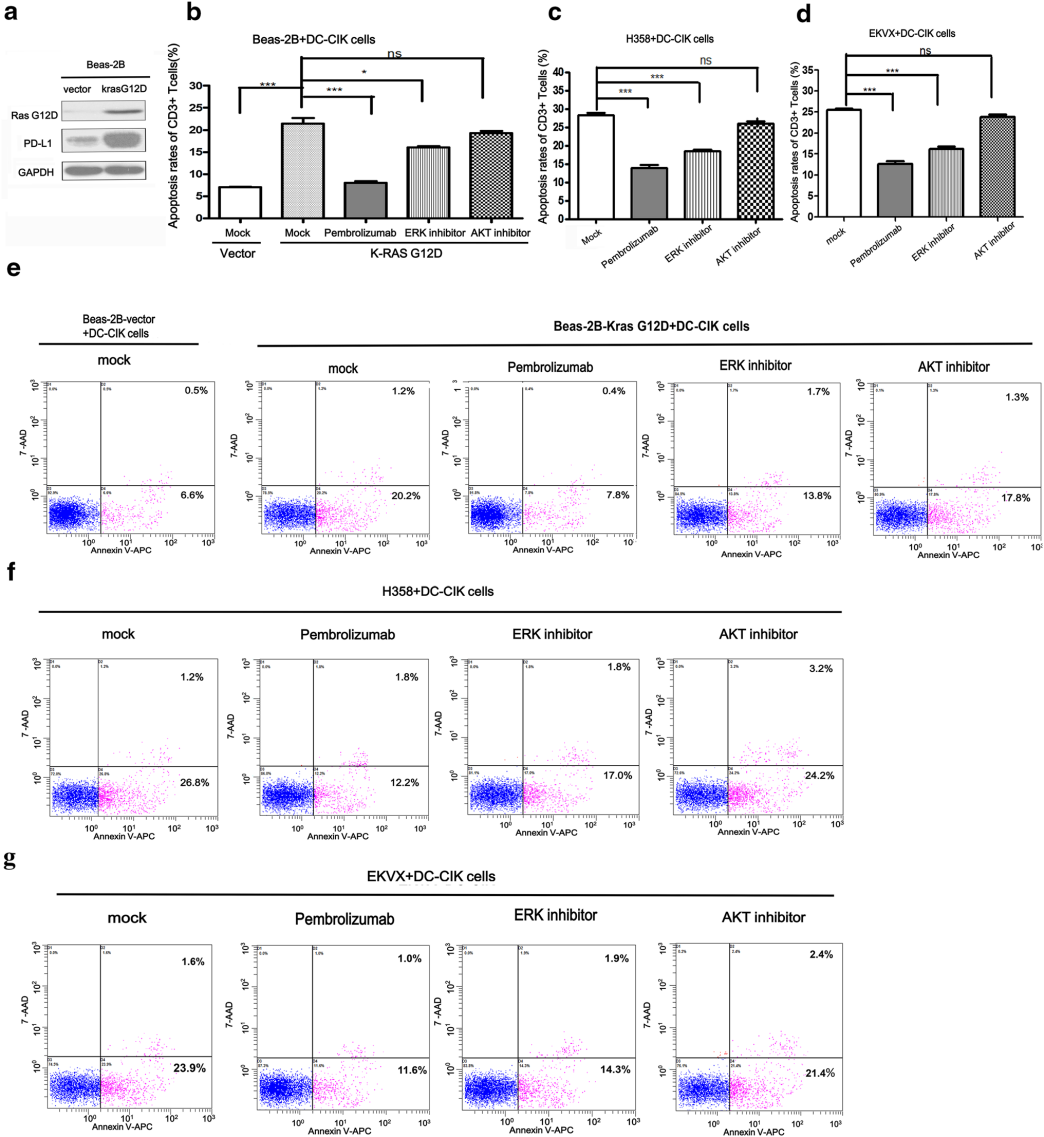
KRAS induced the apoptosis of CD3+ T cells through PD-L1/PD-1 axis and blocking PD-1/PD-L1 could reverse the process. **a** The protein expression level of KRAS-G12D and PD-L1 in Beas-2B-vector and Beas-2B-KRAS G12D cells were detected by western blot. Vector represented the control plasmid of KRAS G12D. Fig. **b**, **c**, **d** is the statistical histogram of Fig. **e**, **f**, **g**. The apoptosis rates of CD3+ T cells were detected by Annexin V-APC/7-AAD apoptosis assay in DC-CIK co-cultured with Beas-2B-vector cells or Beas-2B-KRAS G12D cells (**e**), DC-CIK/H358 co-culture system (**f**) or DC-CIK/EKVX co-culture system (**g**) which were respectively treated with mock (*negative control*), Pembrolizumab (500 μg/ml), ERK1/2 inhibitor (100 nM/L) and AKT inhibitor (1.0 μM/L). The Annexin V-APC-positive cells (both 7-AAD-negative and -positive) were defined as apoptotic cells. Representative results from three independent experiments are shown. *P < 0.05; **P < 0.001; ***P < 0.0001; *ns* indicated as no statistical significance

PD-L1 could lead to apoptosis of T cells, and anti-PD-1 antibody could reverse this process. To investigate whether up-regulation of PD-L1 mediated by KRAS could lead to the apoptosis of T cells, we firstly constructed the stable cell line with KRAS G12D over-expression (Beas-2B-KRAS-G12D) (Fig. 4a). DC-CIK was prepared from peripheral blood donated by healthy volunteers and co-cultured with Beas-2B-KRAS-G12D cells or Beas-2B-vector cells at the ratio of 1:1. As shown in Fig. 4b, e, the apoptosis rates of CD3+ T cells co-cultured with Beas-2B-KRAS G12D cells were higher than that with Beas-2B-vector cells (21.40 ± 1.33 vs. 7.07 ± 0.09%, *P* = 0.0004). On the contrary, blockade of PD-1 on T cells with Pembrolizumab in Beas-2B-KRAS G12D/DC-CIK co-culture system reduced the apoptosis rates of CD3+ T cells to 8.00 ± 0.36% (*P* = 0.0006). Next, we used ERK inhibitor to investigate whether it could reverse the apoptosis of T cells through inhibiting the expression of PD-L1. As expected, the apoptosis rates of CD3+ T cells decreased from 21.40 ± 1.33 to 16.03 ± 0.27% (*P* = 0.0167). However, AKT inhibitor could not reduce the apoptosis rates of T cells compared with mock group (19.23 ± 0.52 vs. 21.40 ± 1.33%, *P* = 0.2037). Next, in H358/DC-CIK co-culture system, ERK inhibitor also reduced the apoptosis rates of CD3+ T cells from 28.30 ± 0.62 to 18.67 ± 0.30% (*P* = 0.0002, Fig. 4c, f). However, the apoptosis rates of CD3+ T cells in the AKT inhibitor group and mock group did not differ (26.10 ± 0.65 vs. 28.30 ± 0.62%, *P* = 0.0713). We also found that the apoptosis rates of CD3+ T cells decreased from 28.30 ± 0.62% in the mock group to 14.03 ± 0.84% in the Pembrolizumab group (*P* = 0.0002). Similarly, we chose EKVX cells (another KRAS-mutant NSCLC cell line) to further confirm that ERK inhibitor and Pembrolizumab could reduce the apoptosis rates of CD3+ T cells (25.47 ± 0.32 vs. 16.17 ± 0.61%, *P* = 0.0002 and 25.47 ± 0.32 vs. 12.60 ± 0.64%, *P* < 0.0001) in EKVX/DC-CIK co-culture system but not with AKT inhibitor (25.47 ± 0.32 vs. 23.83 ± 0.55%, *P* = 0.0616) (Fig. 4d, g). To understand whether the inhibitors and Pembrolizumab may directly affect the T cells, we tested the effects of the two inhibitors and Pembrolizumab on T cells alone. We found that Pembrolizumab (500 μg/ml), ERK1/2 inhibitor (100 nM/L) and AKT inhibitor (1 μM/L) could not influence the viability of DC-CIK and the apoptosis of CD3+ T cells (Supplementary Fig. 2).Taken together, our results demonstrated that KRAS-mediated up-regulation of PD-L1 could induce the apoptosis of CD3+ T cells through PD-1/PD-L1 axis while blocking PD-1 or inhibiting ERK pathway could alleviate the apoptosis of CD3+ T cells.

### Blocking PD-1/PD-L1 axis decreased the survival of KRAS-mutant cells of lung adenocarcinoma in co-culture system

**Fig. 5:**
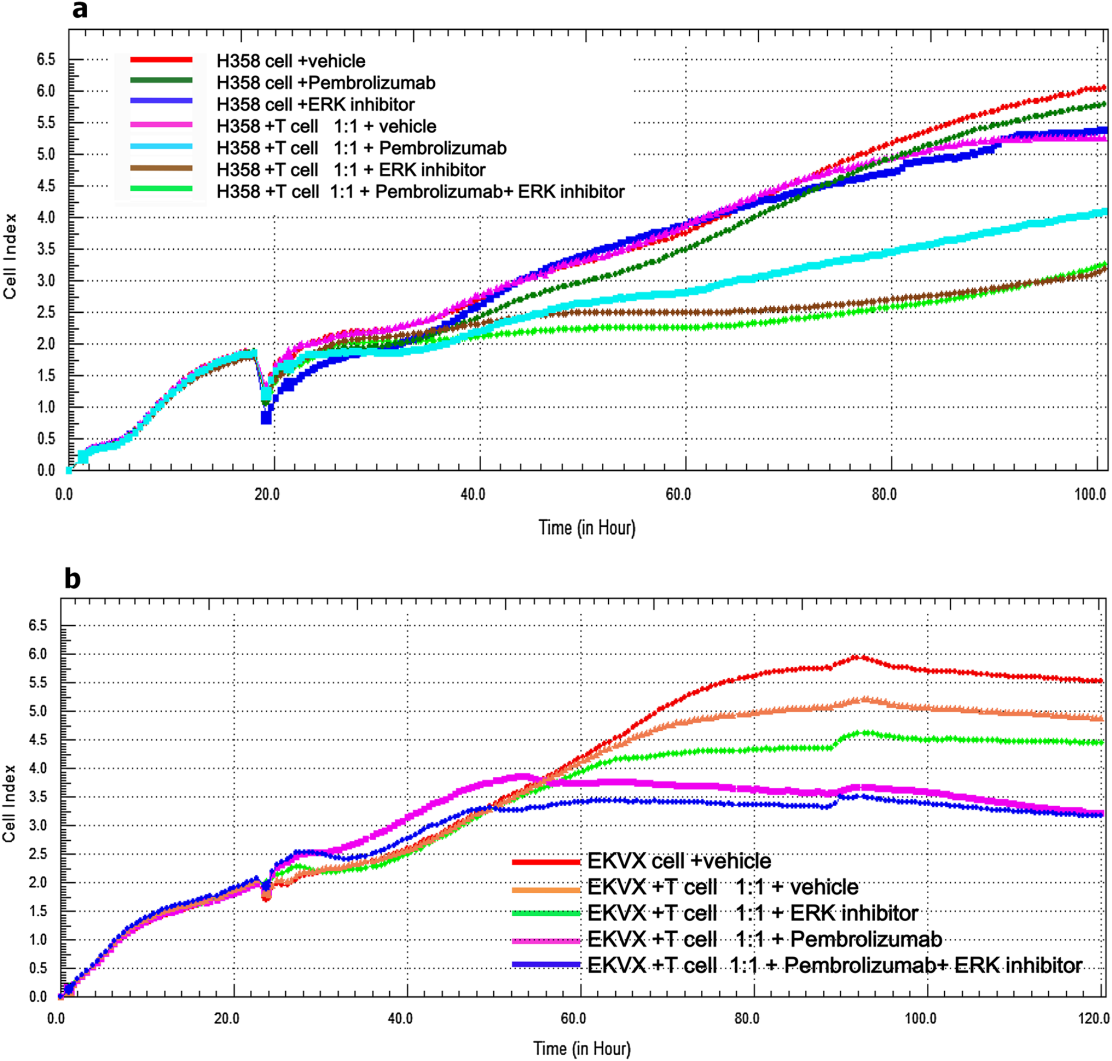
The real time survival curve of KRAS mutant tumor cells (H358 and EKVX) in the co-culture system DC-CIK/H358 (**a**) and DC-CIK/EKVX (**b**) co-culture system were treated with vehicle (PBS), Pembrolizumab (500 μg/ml), ERK inhibitor (100 nM/L) or both. *Cell index* represents the cell proliferation index

Further we examined the real-time survival signal of attached tumor cells after blockade of PD-1 by anti-PD-1 antibody or inhibiting PD-L1 by ERK inhibitor in tumor cells/DC-CIK co-culture system. The cell index represents the survival rate of attached tumor cells excluding the interference of suspended DC-CIK. When H358 cells alone was treated with Pembrolizumab (500 μg/ml), ERK inhibitor (100 nM/L) or vehicle, the cell index of H358 was not obviously changed. However, when H358 or EKVX cells, respectively, co-cultured with DC-CIK at the ratio of 1:1, the survival rates of H358 and EKVX cells decreased in the presence of Pembrolizumab (500 μg/ml), ERK inhibitor (100 nM/L) or both. But combined treatment of Pembrolizumab and ERK inhibitor did not show synergistic tumor-suppression effect (Fig. 5a, b). These results demonstrated that anti-PD-1 antibody or ERK inhibitor could recover the anti-tumor immunity of T cells and therefore decrease the survival rates of KRAS-mutant cells of lung adenocarcinoma in co-culture system.

## Discussion

At present, increasing evidences show that immune suppressive microenvironment is involved in the progression of tumor. The PD-1/PD-L1 axis is an important immune inhibitory pathway contributing to immune escape of cancer cells. It is reported that oncogenes and multiple proinflammatory molecules could regulate PD-L1 [25]. PD-L1 over-expression is more frequently observed in oncogene-addicted lung adenocarcinoma, especially with coexisting mutation subtypes [26]. Our previous studies showed EGFR mutation and ALK rearrangement were associated with PD-L1 expression [15, 16, 27]. Whether PD-L1 was regulated by other driver mutation in NSCLC and its molecular mechanism were largely unknown. In the present study, we demonstrated that PD-L1 expression was positively correlated with KRAS mutation both in the cell lines and tissue of lung adenocarcinoma.

KRAS mutations are detected in approximately 20–25% of lung adenocarcinoma and 4% of squamous cell lung carcinoma [4, 8]. Here, we found a greater proportion of KRAS mutation subgroup showed the higher expression of PD-L1, compared with that of EGFR/ALK/KRAS wild-type subgroup in lung adenocarcinoma patients. However, Calles A et al. recently stated PD-L1 expression was not genetically driven by KRAS mutation but induced by smoking [28]. The explanations for this discrepancy are: firstly, the sensitivity and specificity of anti-PD-L1 antibody affects the expression intensity of PD-L1 in immunohistochemistry experiment. We employed E1L3N clone to evaluate PD-L1 expression not only on tumor cells but also on tumor-infiltrating immune cells, whereas they used clone 9A11 to examine PD-L1 only on tumor cells. Mahoney et al. have reported clone E1L3N may be more sensitive than clone 9A11 in immunohistochemistry [29]. Moreover, the two studies used different cohorts of Eastern and Western patients. Racial difference may play a crucial role in the controversial results. Similar to our finding, recent research of Skoulidis demonstrated lung adenocarcinoma with KRAS mutation and TP53 alteration displayed higher global mutation rates and expressed higher levels of PD-L1 [30]. Also, some studies reported oncogene activation could induce PD-L1 expression which represents the innate immune resistance. For example, constitutive activation of NPM-ALK induced PD-L1 expression in lymphoma [31]. And up-regulation of PD-L1 expression was associated with EGFR mutation and EML4-ALK rearrangements in NSCLC [15, 16, 27]. In addition, PD-L1 expression is often induced by inflammatory factor mainly IFN-γ, which is adaptive immune resistance [18].

To explore the molecular mechanism of PD-L1 up-regulation by KRAS mutation, we tested the downstream signaling pathways of KRAS. Mutant KRAS promotes persistent activation of downstream effectors, leading to survival and proliferation of cancer cells mainly through the Ras/Raf/MEK/ERK pathway or PI3K/AKT pathway. Our study showed that KRAS regulated PD-L1 through p-ERK but not p-AKT signaling. Our previous studies indicated that p-ERK1/2/p-c-Jun but not p-AKT/p-S6 pathway played a critical role in remodeling the expression of PD-L1 regulated by EGFR [15]. P-ERK1/2/p-c-Jun pathway activation was also reported to mediate up-regulation of PD-L1 in BRAF-inhibitor-resistant myeloma [32].

A previous study showed that up-regulation of PD-L1 by EGFR activation mediated the immune escape in EGFR-driven NSCLC [15, 33]. In the present study, we found that up-regulation of PD-L1 by KRAS mutation induced the apoptosis of CD3+ T cells through the PD-1/PD-L1 axis. CD3+ T cells represent the major sub-population of T cells and the apoptosis of CD3+ T cells forebode the anergy and exhaustion of T cells, which results in the immune escape of NSCLC cells.

PD-1/PD-L1 axis is regarded as an important inhibitory pathway in the immune system that is crucial for maintaining self-tolerance and preventing excessive and potentially deleterious T-cell activity [34]. Therapies targeting PD-1 such as Pembrolizumab and Nivolumab have recently shown encouraging efficacy in specific subpopulation of patients with NSCLC [13, 35–37]. We found that Pembrolizumab could re-activate the anti-tumor immunity of T cells and decrease the survival rates of NSCLC cells with endogenous KRAS mutation in co-culture system. Likewise, ERK inhibitor which down-regulated the PD-L1 expression unleashed the T cells’ potence to kill KRAS-mutant tumor cells in the co-culture system but 100 nM ERK inhibitor itself could not affect the survival of H358 cells. We did not observe synergistic effect on killing tumor cells with combination of Pembrolizumab and ERK inhibitor in vitro co-culture system, probably because blocking PD-1 by Pembrolizumab and inhibiting PD-L1 by ERK inhibitor disrupted the same immune pathway of PD-1/PD-L1 axis. Nevertheless, blocking PD-1/PD-L1 axis provides a promising strategy for KRAS-mutant NSCLC with PD-L1 up-regulation. Recently, Davar reported a patient with advanced, heavily pretreated KRAS-mutant lung adenocarcinoma who developed an excellent response after a single-dose of anti-PD-1 antibody (nivolumab) [38].

In conclusion, our study found PD-L1 expression is correlated with KRAS mutation in lung adenocarcinoma. Furthermore, we clarified that PD-L1 was up-regulated by KRAS over-expression through p-ERK but not p-AKT signaling. We also found KRAS-mediated up-regulation of PD-L1 induced the apoptosis of CD3+ T cells and mediated immune escape in lung adenocarcinoma cells, which could be reversed by anti-PD-1 antibody or ERK inhibitor treatment. Pembrolizumab or ERK inhibitor could recover the anti-tumor immunity of T cells and decrease the survival rates of KRAS-mutant NSCLC cells in co-culture system in vitro. Our study might provide a promising therapeutic option for NSCLC with KRAS mutation.

## Electronic supplementary material

Below is the link to the electronic supplementary material.

Supplementary material 1 (PDF 543 kb)

## Abbreviations

ALK: Anaplastic lymphoma kinase
CST: Cell signaling technology
CTLA-4: Cytotoxic T lymphocyte associated antigen-4
DC-CIK: Dendritic cells and cytokine-induced killer cells
EGFR: Epidermal growth factor receptor
EML4: Echinoderm microtubule associated protein like 4
KRAS: Kirsten rat sarcoma viral oncogene homolog
NSCLC: Non-small-cell lung cancer
PD-1: Programmed death-1 receptor
PD-L1: Programmed death ligand 1
TKIs: Tyrosine kinase inhibitors
