# Supplementary material for: KRAS mutation-induced upregulation of PD-L1 mediates immune escape in human lung adenocarcinoma
Source: Cancer Immunol Immunother. 2017 Apr 27;66(9):1175–87. doi: 10.1007/s00262-017-2005-z (PMC5579171; doi:10.1007/s00262-017-2005-z)
Supplement: Supplementary file 1 — Supplementary material 1 (PDF 543 kb) [file 262_2017_2005_MOESM1_ESM.pdf]

Supplementary figure 1

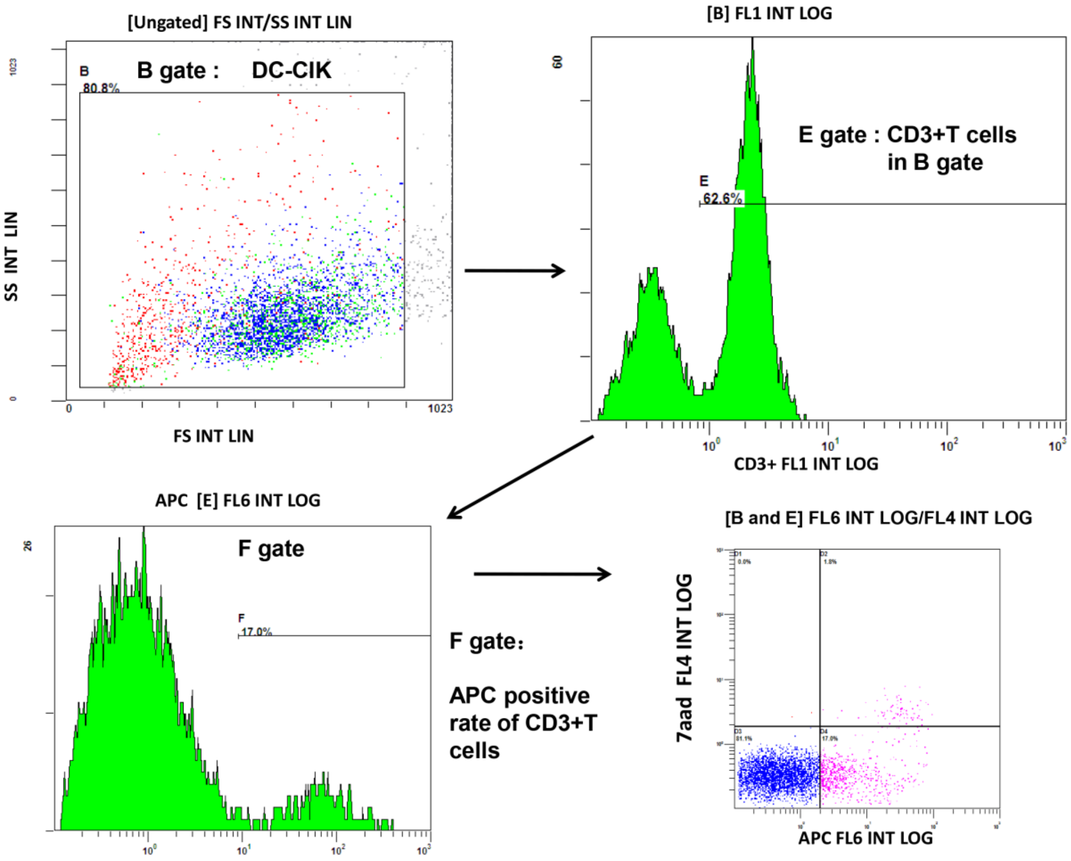

Supplementary figure 1 | gating strategy for selecting CD3+ T cells from DC-CIK cells

Supplementary Figure 2

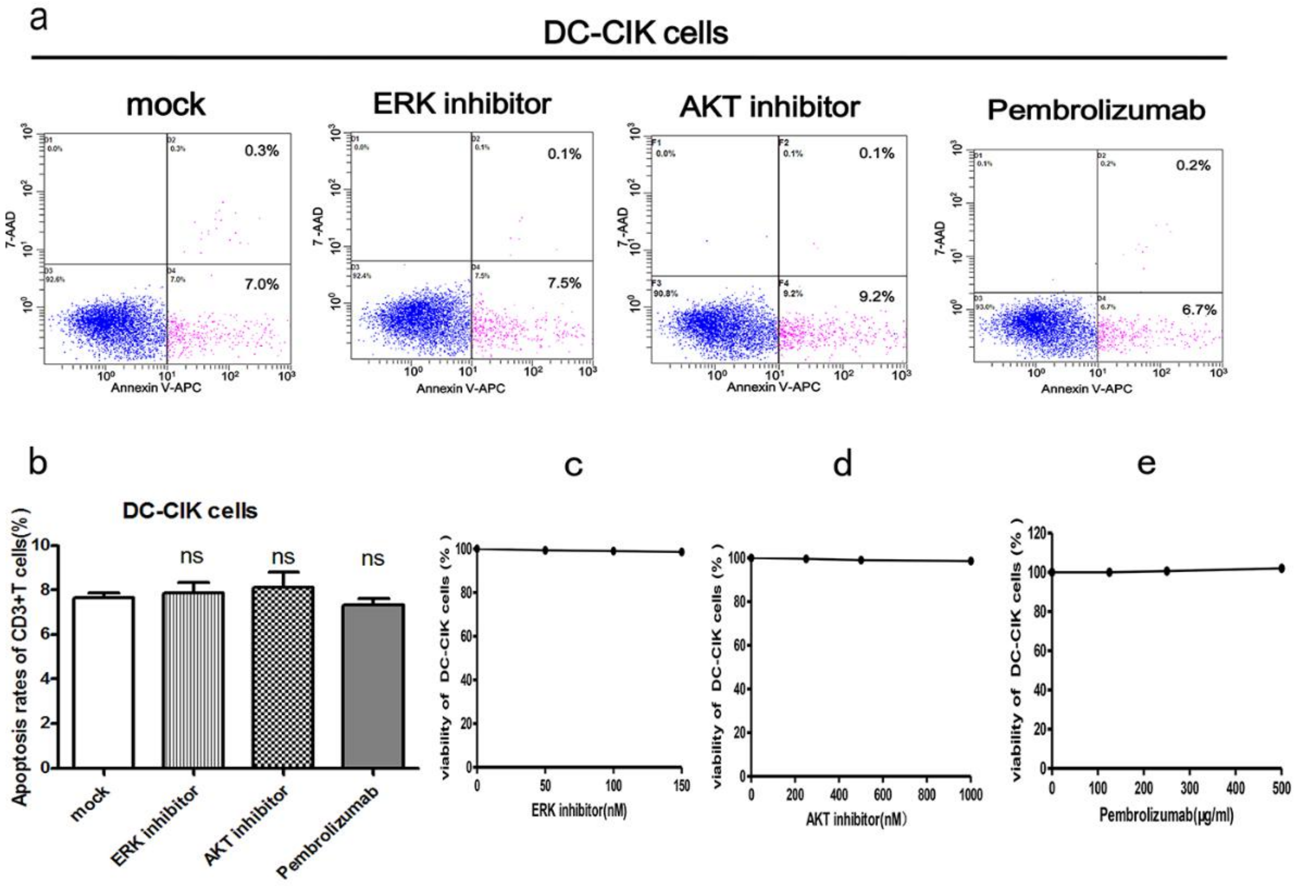

Supplementary figure 2 | Annexin V-APC/7-AAD apoptosis assay and cell viability assay with CCK8 kit. **a** The apoptosis rates of CD3+T cells from DC-CIK cells which were treated with mock (PBS), ERK inhibitor (100 nM/L), AKT inhibitor (1.0 μM/L) or pembrolizumab (500 μg/ml) for 48h. **b** Statistical histogram of (a). **c, d, e** the viability of DC-CIK cells when treated with different concentrations of ERK inhibitor (0, 50, 100, 150 nM/L) (**c**), AKT inhibitor (0, 250, 500, 1000 nM/L) (**d**) or pembrolizumab (0, 125, 250, 500 μg/ml) (**e**) for 72h. Representative results from three independent experiments are shown. “ns” indicated as no statistical significance.
